# Effect of generation rate on transient photoconductivity of semi-insulating 4H-SiC

**DOI:** 10.1038/s41598-020-68898-z

**Published:** 2020-07-17

**Authors:** Marek Suproniuk, Mariusz Wierzbowski, Piotr Paziewski

**Affiliations:** Department of Electronics, Military University of Technology, Gen. Kaliskiego Str. 2, 00-908 Warsaw 46, Poland

**Keywords:** Electronic devices, Characterization and analytical techniques

## Abstract

The effect of generation rate on transient photoconductivity of semi-insulating (SI) 4H-SiC is discussed. The rate of generation of electron–hole pairs is dependent on the number of photons incident on the sample material and its absorption and reflection coefficients. The number of photons and their energy is dependent on the radiation power and wavelength of the light source illuminating the material. The results of research, obtained with a specialized simulator, present the influence of changes in the filling of individual defect centres’ levels on changes in conductivity of the test material observed after switching on the photoexcitation. For the purpose of simulations, presented is a versatile model of semiconductor material. It encompasses six point defects that appear in SI 4H-SiC materials the most often. Those defect centres correspond to Z_1/2_ recombination centre, deep electron and deep hole traps, nitrogen-related shallow donors of two kinds and a boron-related shallow acceptor. The simulation results can be used to design and determine properties of photoconductive switches.

## Introduction

In recent years, there is intensive research being conducted which aims at developing semiconductor materials with new properties that allow to design devices for new system solutions in power electronics. The new material properties are obtained with defect structure engineering, which involves introducing defect centres with certain properties into a semiconductor material. Currently, research is focused on providing materials for a new type of devices which can operate in a broader temperature range and at higher frequencies while withstanding both higher current densities and higher electric fields. An important group of the materials are semi-insulating monocrystals, characterized with resistivities above 10^5^ Ω cm, which are intended for use in microelectronics as substrates for new generation of integrated circuits and in electroenergetics as bulk materials for photoconductive semiconductor switches (PCSSes)^1,2^.

Currently, one of the most widely used materials for the application is semi-insulating silicon carbide (SI SiC)^3^. Its properties are desired in electroenergetics for manufacturing hybrid switches^4^. Additionally, its wide band gap enables devices based one the semi-insulating silicon carbide to operate in a range of temperatures up to 600 °C. Designing such devices requires an efficient method of investigation of the material electrical properties. One of the most often used method is photo-induced transient spectroscopy (PITS)^5,6^. The method is intended for investigating defect structure of semiconductor materials and involves filling defect centres’ levels with excess electrons or holes generated during illumination of the test material with a pulse of light and then measuring the transient waveform of photocurrent relaxation induced by thermal emission of charge carriers after turning the photoexcitation off^5,6^. As the principle of operation of the photoconductive switches is based on the photoconductivity phenomenon^1^, the PITS method is a very useful method for investigating the switches’ properties as well. In this context, simulation studies of rates of changes in excess charge carrier concentrations are becoming very important for selection of measurement conditions that provide a proper quality of measured signals^7,8^. Currently available kinetics models of the transient photoconductivity phenomenon in semiconductor materials^9–14^ include only small number of defect centres, omitting the recombination centres. We exploit a model described by a system of differential equations proposed in our earlier works^15–17^, which is more complex as it enables us to simulate the kinetics phenomena in the presence of three types of defect centres, i.e. donor-like electron traps, acceptor-like hole traps and a donor-like recombination centre. Moreover, it can be easily modified and extended with successive defect centres, including other recombination centres^7,8^, and with other optical phenomena.

In this paper we present an applicable model of semi-insulating (SI) 4H-SiC, which is employed to simulations of photoconductivity changes induced by illuminating the material with light of various optical power. The simulations let us investigate how the illumination conditions like the power (expressed here in the form of generation rate) affect dynamics of the photoconductivity changes and what the role of individual defect centres in this process is. The knowledge is crucial in terms of designing the PCSSes^18^ as the optical properties of the device’s material (induced by generation and recombination processes of charge carriers) determine its turn-on and turn-off times and consequently its maximum switching frequency.

## Methods

### Modelling the photo-induced processes of charge carrier generation

Illumination of the semiconductor material with a photon flux Φ of energy greater than the band-gap width (*h*v > *E*_*g*_) causes absorption of photons and generation of additional electron–hole pairs. As a result of the generation, an increase in the concentration of the excess electrons in the conduction band and the excess holes in the valence band takes place. The rate of the electron–hole pairs generation as a function of the depth from the surface of the illuminated sample material can be expressed by the following formula^19^:

1 $$G\left( x \right) = G_{s} e^{{ - \alpha x}},$$

where *G*_*s*_ is the rate of generation of the electron–hole pairs in the subsurface region of the sample and is determined with the expression:

2 $$G_{s} = \eta \alpha \left( {1 - R} \right)\frac{{\lambda P_{0} }}{{hcA}},$$

where η is the quantum efficiency of the electron–hole pairs generation, α—the absorption coefficient, λ—the wavelength of the source light, *R*—the reflection coefficient, *A*—the field of the illuminated surface, *P*_0_—the power of the source light, *h*—Planck's constant and *c*—the speed of light in a vacuum. Assuming a uniform illumination of the sample material, the photon flux is directly proportional to the number of photons *N*_*ph*_ incident on the surface per unit time and inversely proportional to the area of the illuminated surface. The number of photons *N*_*ph*_ depends on the power of the light beam and is given by:

3 $$N_{{ph}} = \frac{{\lambda P_{0} }}{{hc}}.$$

By substituting the Eq. (3) in (2), it is easy to notice that the rate of the generation of the excess charge carriers is proportional to the photon flux.

For the simulation is conducted in zero-dimensional space (we don’t take any spatial variables into consideration), an average rate of generation of electron–hole pairs must be determined. It is obtained by integrating the Eq. (1) over the range of depth of photon penetration:

4 $$G_{{avg}} = \alpha \int\limits_{0}^{{\frac{1}{\alpha }}} {G_{s} e^{{ - \alpha x}} {\text{d}}x} .$$

The integration range results from the assumption that thickness of the sample material *b* = 300 μm is much greater than the range of penetration depth of photons. In our case, i.e. for λ = 375 nm, the absorption coefficient α = 3 × 10^4^ cm^−1^ and the product α*b* = 900 ≫ 1, thus the depth can be estimated as 1/α^20^. We also assume the following parameters: η = 1, *R* = 0.3, *c* = 2.998 × 10^8^ m/s, *h* = 6.626 × 10^–34^ Js, *A* = π*d*^2^/4, where *d* is diameter of a circular-shaped spot of laser light incident between ohmic contacts of the sample material and equals exactly the distance between the ohmic contacts *d* = 0.07 cm. Given the exemplary value of the light beam power *P*_0_ = 5 mW, that illuminates the sample material, the calculated average rate of the generation is equal to *G*_*avg*_ = 3.256 × 10^22^ cm^−3^ s^−1^.

### Material properties

The impact of the generation rate *G* of electron–hole pairs on changes in photoconductivity results from dynamics of changes in degree of filling the individual defect centres with charge carriers generated by incident photons. Study of the impact has been performed based on our model of the 4H-SiC material^17,21^. The model assumes existence of six defect centres: Z_1/2_ recombination centre, deep electron and deep hole traps, nitrogen-related shallow donors of two kinds and a boron-related shallow acceptor. Parameters of the defect centres were adopted from^22,23^ obtained as a result of experimental investigation.

In the model, two shallow donors are associated with the occurrence of nitrogen atoms which replace carbon atoms present in two configurations, hexagonal (*h*) and cubic (*k*) sites of the 4H-SiC crystal lattice. Shallow acceptors are boron atoms replacing the silicon atoms present in both the hexagonal (*h*), and the cubic (*k*) sites. For the electron trap, the ID9 centre as a native defect observed in SiC is assumed, while for the hole trap, the HK3 centre observed with the electron paramagnetic resonance (EPR) method^22^, was assumed. Properties of the recombination centre are assumed to be identical with the properties of the Z_1/2_ centre. The Z_1/2_ centres are very characteristic of the material and probably are associated with double vacancies: a lack of carbon in *h* site and a lack of silicon in *k* site or a lack of carbon in *k* site and a lack of silicon in *h* site^23,24^. Additionally, we assumed the presence of another type of recombination process which is a band-to-band direct recombination. Parameter values of each defect centre are shown in Table 1^16,17^.

The transient photoconductivity induced by the generation of electron–hole pairs by the flux of photons with energy of *hv* > *E*_*g*_ is given by:

5 $$\sigma \left( t \right) = q\left[ {n\left( t \right) \cdot \mu_{n} + p\left( t \right) \cdot \mu_{p} } \right],$$

where *q* is the elementary charge, µ_*n*_ = 950 cm^2^ V^−1^ s^−1^ and µ_*p*_ = 150 cm^2^ V^−1^ s^−1^^20^ are mobilities of electrons and holes, respectively, and *n*(*t*) and *p*(*t*) are concentrations of excess electrons and holes, respectively. The concentrations may be determined by solving a set of rate equations that describe the electronic transitions as the rate of change in concentration of electrons in the conduction band and of holes in the valence band, as well as the rate of change in concentration of electrons and holes filling each of the defects. Due to the types of defects present in the assumed SI 4H-SiC material, considered were phenomena like defect-centre-based carrier capture and emission, and direct band-to-band recombination. In result, the equations, adopted from our previous work^17^ and extended with the new type of recombination process, can now be defined as:

6a $$\begin{aligned} \frac{dn\left( t \right)}{{dt}} & = \underbrace {{e_{SD1} n_{SD1} \left( t \right) - n\left( t \right)c_{SD1} \left( {\frac{{N_{SD} }}{2} - n_{SD1} \left( t \right)} \right)}}_{SD1} + \underbrace {{e_{SD2} n_{SD2} \left( t \right) - n\left( t \right)c_{SD2} \left( {\frac{{N_{SD} }}{2} - n_{SD2} \left( t \right)} \right)}}_{SD2} \\ & \quad + \underbrace {{e_{ET} n_{ET} \left( t \right) - n\left( t \right)c_{ET} \left( {N_{ET} - n_{ET} \left( t \right)} \right)}}_{ET} + \underbrace {{e_{{RC_{n} }} n_{RC} \left( t \right) - n\left( t \right)c_{{RC_{n} }} \left( {N_{RC} - n_{RC} \left( t \right)} \right)}}_{RC} - \frac{n\left( t \right)}{\tau } + G, \\ \end{aligned}$$

6b $$\begin{aligned} \frac{dp\left( t \right)}{{dt}} & = \underbrace {{e_{RCp} \left( {N_{RC} - n_{RC} \left( t \right)} \right) - p\left( t \right)c_{RCp} n_{RC} \left( t \right)}}_{RC} + \underbrace {{e_{HT} \left( {N_{HT} - n_{HT} \left( t \right)} \right) - p\left( t \right)c_{HT} n_{HT} \left( t \right)}}_{HT} \\ & \quad + \underbrace {{e_{SA} \left( {N_{SA} - n_{SA} \left( t \right)} \right) - p\left( t \right)c_{SA} n_{SA} \left( t \right)}}_{SA} - \frac{p\left( t \right)}{\tau } + G, \\ \end{aligned}$$

6c $$\frac{{dn_{SD1} \left( t \right)}}{dt} = - \underbrace {{e_{SD1} n_{SD1} \left( t \right)}}_{n\,emission} + \underbrace {{n\left( t \right)c_{SD1} \left( {\frac{{N_{SD} }}{2} - n_{SD1} \left( t \right)} \right)}}_{n\,capture},$$

6d $$\frac{{dn_{SD2} \left( t \right)}}{dt} = - \underbrace {{e_{SD2} n_{SD2} \left( t \right)}}_{n\,emission} + \underbrace {{n\left( t \right)c_{SD2} \left( {\frac{{N_{SD} }}{2} - n_{SD2} \left( t \right)} \right)}}_{n\,capture},$$

6e $$\frac{{dn_{ET} \left( t \right)}}{dt} = - \underbrace {{e_{ET} n_{ET} \left( t \right)}}_{n\,emission} + \underbrace {{n\left( t \right)c_{ET} \left( {N_{ET} - n_{ET} \left( t \right)} \right)}}_{n\,capture},$$

6f $$\frac{{dn_{RC} \left( t \right)}}{dt} = - \underbrace {{e_{RCn} n_{RC} \left( t \right)}}_{n\,emission} + \underbrace {{n\left( t \right)c_{RCn} \left( {N_{RC} - n_{RC} \left( t \right)} \right)}}_{n\,capture} + \underbrace {{e_{RCp} \left( {N_{RC} - n_{RC} \left( t \right)} \right)}}_{p\,emission} - \underbrace {{p\left( t \right)c_{RCp} n_{RC} \left( t \right)}}_{p\,capture},$$

6g $$\frac{{dn_{HT} \left( t \right)}}{dt} = \underbrace {{e_{HT} \left( {N_{HT} - n_{HT} \left( t \right)} \right)}}_{p\,emission} - \underbrace {{p\left( t \right)c_{HT} n_{HT} \left( t \right)}}_{p\,capture},$$

6h $$\frac{{dn_{SA} \left( t \right)}}{dt} = \underbrace {{e_{SA} \left( {N_{SA} - n_{SA} \left( t \right)} \right)}}_{p\,emission} - \underbrace {{p\left( t \right)c_{SA} n_{SA} \left( t \right)}}_{p\,capture},$$

where parameter *G* is the direct (band-to-band) generation rate for electron–hole pairs, *e*_*SD*1_ and *e*_*SD*2_, are thermal emission rates of electrons for type 1 and type 2 shallow donors, respectively, *e*_*ET*_ and *e*_*RCn*_ are thermal emission rates for electron traps and for recombination centres, respectively, and *e*_*SA*_, *e*_*HT*_ and *e*_*RCp*_ are thermal emission rates of holes for shallow acceptors, hole traps and recombination centres, respectively, while capture coefficients of electrons for according defect centres are denoted by *c*_*SD*1_, *c*_*SD*2_, *c*_*ET*_ and *c*_*RCn*_, respectively, and finally, capture coefficients of holes for according defect centres are denoted by *c*_*SA*_, *c*_*HT*_ and *c*_*RCp*_, respectively.

Changes in carrier concentration in the bands, resulting from exchanging electrons between the valence and conduction bands, are dependent on three factors: (I) generation rate of charge carriers induced by the incident light, (II) coefficients of thermal emission and capture of carriers associated with defect levels and (III) carrier lifetime associated with direct (band-to-band) recombination. The thermal emission rate *e*_*n*_ of electrons from defect centres to the conduction band and the thermal emission rate *e*_*p*_ of holes to the valence band are calculated from the Arrhenius equation:

7a $$e_{n} = \sigma_{n} \cdot \gamma_{n} \cdot T^{2} \cdot \exp \left( {\frac{{ - E_{an} }}{{k_{B} \cdot T}}} \right),$$

7b $$e_{p} = \sigma_{p} \cdot \gamma_{p} \cdot T^{2} \cdot \exp \left( {\frac{{ - E_{ap} }}{{k_{B} \cdot T}}} \right),$$

where σ_*n*_ and σ_*p*_ denote capture cross-sections for electrons and holes, accordingly, *T* is the absolute temperature assumed to be 300 K, *E*_*an*_ is activation energy of electron emission from a particular type of defect centre with respect to bottom of the conduction band, whereas similarly, *E*_*ap*_ is activation energy of hole emission from a particular type of defect centre in relation to top of the valence band, γ_*n*_ and γ_*p*_ coefficients, determined by effective masses of electrons and holes, are equal to 2.5 × 10^21^ and 3.26 × 10^21^ cm^−2^ s^−1^ K^−2^^19^, respectively, and *k*_*B*_ is Boltzmann’s constant. The capture rates for a given type of defect centre are represented by capture coefficients *c*_*n*_ and *c*_*p*_ for electrons and holes, respectively, and are defined as:

8a $$c_{n} = \sigma_{n} \cdot v_{n} ,$$

8b $$c_{p} = \sigma_{p} \cdot v_{p} ,$$

where *v*_*n*_ = 7.685 × 10^5^*T*^0.5^ cm^1 ^s^−1^ and *v*_*p*_ = 6.744 × 10^5^*T*^0.5^ cm^1^ s^−1^^19^ are average electron and hole thermal speeds, respectively, and are dependent on the effective mass and the temperature. The lifetime parameter associated with the rates of direct band-to-band recombination processes of electrons and holes was assumed to correspond to electron lifetime related to transition of electrons to the recombination centre level and was obtained with:

9 $$\tau = \frac{1}{{N_{RC} \cdot \sigma_{n} \cdot v_{n} }} .$$

In Table 2 we present coefficients determined with the Eqs. (7)–(9) for all the defect centres.

**Table 1:** Properties of defect centres applied for simulation of kinetics of 4H-SiC material^16, 17^.

Defect label	Defect type	Activation energy [eV]	Concentration [cm^−3^]	Capture cross-section [cm^2^]		Identification
				Electrons	Holes	
SD1	Shallow donor	*E*_*c*_ − 0.050	5 × 10^15^	~ 4.0 × 10^–20^	–	N_C_ in *h* site^24^
SD2	Shallow donor	*E*_*c*_ − 0.092	5 × 10^15^	~ 4.0 × 10^–19^	–	N_C_ in *k* site^24^
ET	Electron trap	*E*_*c*_ − 0.555	5 × 10^14^	~ 1.2 × 10^–15^	–	ID9^24^
RC	Recombination centre	*E*_*c*_ − 0.630	8 × 10^14^	1.3 × 10^–14^	1.0 × 10^–14^	Z_1/2_ centre^23, 24^
HT	Hole trap	*E*_*v*_ + 1.125	7 × 10^14^	–	~ 3.1 × 10^–14^	ID centre^22^, HK3 trap^26^
SA	Shallow acceptor	*E*_*v*_ + 0.285	9.7 × 10^15^	–	~ 3.1 × 10^–17^	B_Si_ in both *h* and *k* sites^24^

**Table 2:** Coefficients assumed for the kinetics simulation of charge carriers exchange rates for each defect centre in 4H-SiC material.

Defect label	Thermal emission [s^−1^]	Capture [cm^3^s^−1^]	Lifetime [s]
SD1	1.2997 × 10^6^ (*e*)	5.2977 × 10^–13^ (*e*)	7.2237 × 10^–9^
SD2	2.5601 × 10^6^ (*e*)	5.2977 × 10^–12^ (*e*)	
ET	126.52 (*e*)	1.5707 × 10^–8^ (*e*)	
RC	76.61 (*e*)/4.9967 × 10^–32^ (*h*)	1.7304 × 10^–7^ (*e*)/1.1681 × 10^–7^ (*h*)	
HT	1.1359 × 10^–6^ (*h*)	3.5861 × 10^–7^ (*h*)	
SA	1.4682 × 10^5^ (*h*)	3.5861 × 10^–10^ (*h*)	

In order to simulate the kinetics of the photo-induced changes in charge carrier concentration, the equilibrium Fermi level and concentration of carriers at trap levels and in the bands must be first calculated. It is accomplished, based on the Fermi–Dirac statistics, by solving the charge neutrality equation for assumed concentrations of defect centres at the temperature of 300 K taken here. The equation describing our model of the 4H-SiC material can be represented by^17^:

10 $$n_{0} - N_{SD1}^{ + } - N_{SD2}^{ + } - N_{ET}^{ + } = p_{0} - N_{SA}^{ - } - N_{RC}^{ - } - N_{HT}^{ - } ,$$

where equilibrium concentrations of electrons and holes are denoted by *n*_0_ and *p*_0_, respectively, whereas concentrations of the ionized donors SD1, SD2, and ET are denoted by $N_{SD1}^{ + }$, $N_{SD2}^{ + }$, and $N_{ET}^{ + }$, respectively, and concentrations of the ionized acceptors SA, HT, and RC are denoted by $N_{SA}^{ - }$, $N_{HT}^{ - }$, and $N_{RC}^{ - }$, respectively. The Fermi level determined with the Eq. (10) equals 2.536 eV.

Finally, by knowing the electron concentration in the conduction band and the hole concentration in the valence band, the conductivity of the semiconductor material in the absence of light can be determined. Additionally, based on the Fermi–Dirac statistics, concentration of electrons filling each energy levels can also be determined. The equilibrium concentrations were assumed as starting conditions (at *t* = 0) for solving the kinetics Eqs. (6) describing phenomena occurring during illumination of the semiconductor material with stream of photons. They are solved numerically with a standard computing environment (MATLAB version R2019b^25^ ran on Microsoft Windows 10) using the Runge–Kutta-Fehlberg integration method with automatically controlled time step size.

## Results

### Impact of the generation rate on transient photoconductivity of SI 4H-SiC

In our paper we focus on simulations of changes in carrier concentrations, which directly influence the material conductivity, and therefore the value of electric current. For a sufficiently high stream of photons, concentration of excess electrons in the conduction band *n*(*t*) and concentration of the excess holes in the valence band *p*(*t*) are much greater than the equilibrium concentrations, i.e. *n*_0_ and *p*_0_, respectively. The simulations of charge carrier kinetics were carried out for several cases of various optical powers. For each of the cases, an identical and perfect light pulse, starting at *t* = 0, with zero rise time and duration of 10 μs, was assumed (usually after this time an equilibrium was reached). Considered were four values of optical power which correspond to four values of electron–hole pairs generation rate coefficient *G* which were based on rounded-up value of *G*_*avg*_, i.e. *G* = 3.3 × 10^20^ cm^−3^ s^−1^, *G* = 3.3 × 10^22^ cm^−3^ s^−1^, *G* = 3.3 × 10^24^ cm^−3^ s^−1^, and *G* = 3.3 × 10^26^ cm^−3^ s^−1^, with the second one related to the power level *P*_0_ = 5 mW.

**Figure 1:**
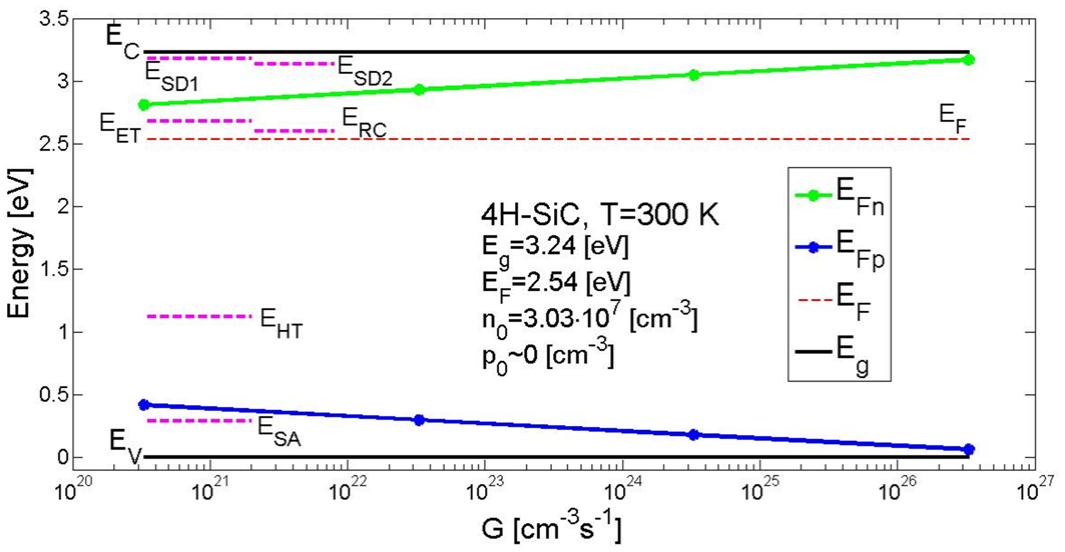
Energy levels in the band gap of SI 4H-SiC (at 300 K). They were assumed for solving the equations that describe rate of change in concentration of excess electrons and holes after a photo-induced generation of electron–hole pairs at rate of *G.*

As a result, determined were temporal changes in carrier concentration in conduction and valence bands, and at the defect levels in band gap for the semiconductor 4H-SiC material at the temperature of 300 K. The concentration of carriers in the bands affects the conductivity of the material. Additionally, the illumination of the material causes division of the Fermi level into two quasi-levels. Figure 1 presents the Fermi levels for each individual value of the electron–hole pairs generation rate coefficients. By increasing the rate of generation the quasi-Fermi level for electrons also increases, from *E*_*Fn*_ = 2.82 eV for *G* = 3.3 × 10^20^ cm^−3^ s^−1^ to *E*_*Fn*_ = 3.18 eV for *G* = 3.3 × 10^26^ cm^−3^ s^−1^ and at the same time the quasi-Fermi level for holes decreases from *E*_*Fp*_ = 0.42 eV for *G* = 3.3 × 10^20^ cm^−3^ s^−1^ to *E*_*Fp*_ = 0.06 eV for *G* = 3.3 × 10^26^ cm^−3^ s^−1^. Additionally, presented are the activation energies of defect centres assumed for the model and the Fermi level, equal 2.536 eV, determined from the electrical neutrality Eq. (5).

**Figure 2:**
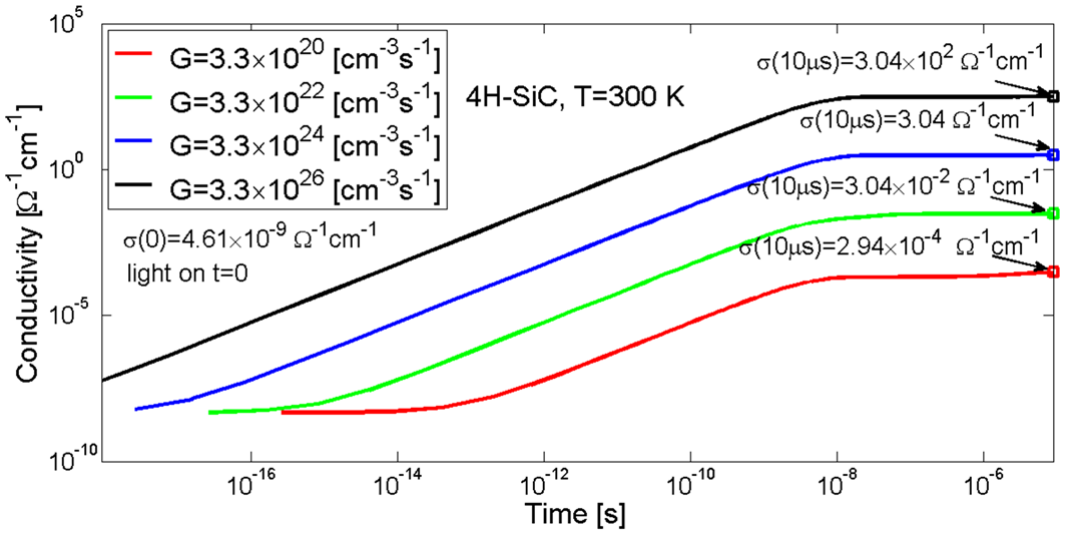
Simulation results of conductivity changes of 4H-SiC material. The changes, caused by photo-induced generation of electron–hole pairs, are shown here for various values of laser light power given as the generation rate coefficient *G.*

Temporal changes in conductivity of the material induced by incident stream of photons are shown in Fig. 2. Before illuminating the material, i.e. in the thermoelectric equilibrium state, the conductivity of the material was equal to σ(0) = 4.62 × 10^–9^ Ω^−1^ cm^−1^. It can be seen that the greater the rate of generation of electron–hole pairs the earlier the change in conductivity of the material appears. For *G* = 3.3 × 10^26^ cm^−3^ s^−1^ the conductance changes are visible as early as at *t* ~ 2.6 × 10^–19^ s, for *G* = 3.3 × 10^24^ cm^−3^ s^−1^ a little bit later, i.e. at *t* ~ 2.6 × 10^–19^ s, for *G* = 3.3 × 10^22^ cm^−3^ s^−1^ even later, i.e. at *t* ~ 2.6 × 10^–17^ s and for *G* = 3.3 × 10^20^ cm^−3^ s^−1^ as late as at *t* ~ 2.6 × 10^–16^ s. Before the time reaches 10 ns, for all the generation rates, a constant increase in conductivity per decade can be seen, and after time of 10 μs it is equal to 3.02 × 10^2^, 3.02 × 10^0^, 3.02 × 10^–2^ and 2.94 × 10^–4^ Ω^−1^ cm^−1^, respectively. The simulations show that for the time of 1 ps for the rate of generation *G* = 3.3 × 10^26^ cm^−3^ s^−1^, the conductance is greater than for the time of 10 μs at the smaller rate of generation *G* = 3.3 × 10^22^ cm^−3^ s^−1^.

**Figure 3:**
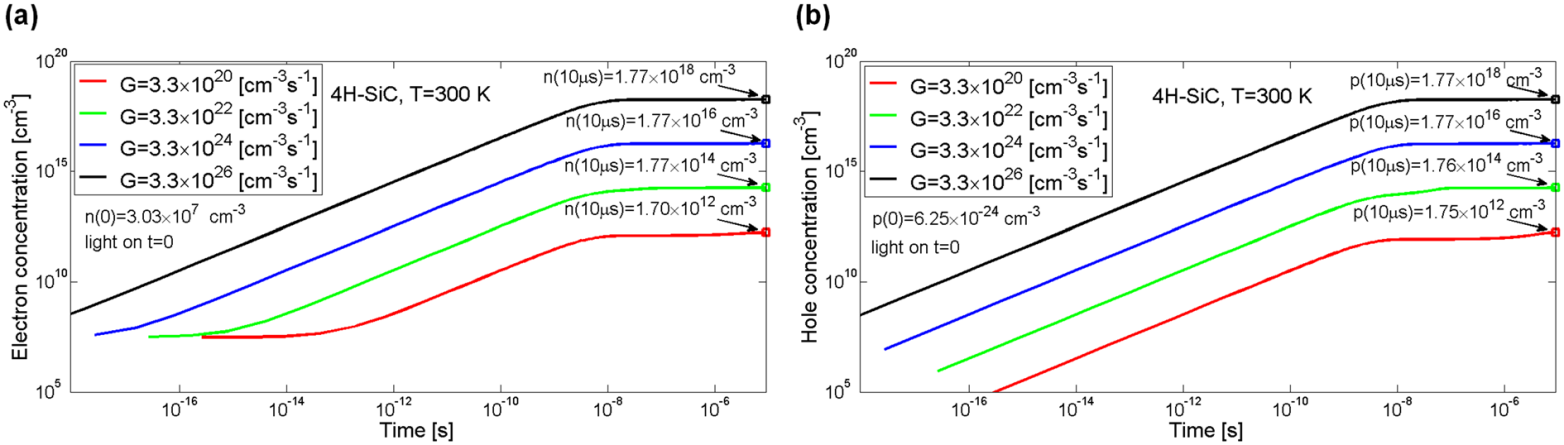
Simulation of temporal changes in electron concentration in the conduction band (**a**) and hole concentration in the valence band (**b**) in 4H-SiC material. Parameter *G* is the generation rate coefficient.

Changes in the photoconductivity shown in Fig. 2 are a result of changes in the concentration of excess charge carriers in the conduction and valence bands, obtained as a solution to the kinetics equations^18^. It can be noticed that regardless from the electron–hole pairs generation rate the conductivity settles after about 10 μs which is related to carrier lifetime. Temporal changes in electron concentration *n*(*t*) for a given set of generation rates of electron–hole pairs *G* is shown in Fig. 3a, whereas likewise changes in hole concentration *p*(*t*) is shown in Fig. 3b. However it should be noted that before the illumination started concentration of electrons in the conduction band was equal to *n*(0) = 3.03 × 10^7^ cm^−3^, while concentration of holes *p*(0) was close to zero.

By comparing the concentrations of holes and electrons after all the transitional processes associated with the kinetics of photoconductivity stopped, i.e. after a time period of 10 µs, it can be stated that increase in concentration of holes in the valence band is about two times greater than the increase in concentration of electrons in the conduction band. However, as it has been already stated, the greater impact on the 4H-SiC material conductivity changes has the concentration of electrons in conduction band rather than the concentration of holes in the valence band since the charge carriers mobility assumed for the model is 6.33 times greater for electrons than for holes. It can also be noticed that increase in the generation rate of electron–hole pairs *G* results in proportional increase in the concentration of electrons in the conduction band as well as holes in the valence band. For electrons the concentrations are equal to 1.66 × 10^18^, 1.66 × 10^16^, 1.66 × 10^14^, and 1.61 × 10^12^ cm^−3^, and for holes the concentrations are equal to 2.46 × 10^18^, 2.46 × 10^16^, 2.45 × 10^14^, 2.44 × 10^12^ cm^−3^, for subsequent values of *G*.

### Impact of the generation rate on the occupation of electron traps in SI 4H-SiC

**Figure 4:**
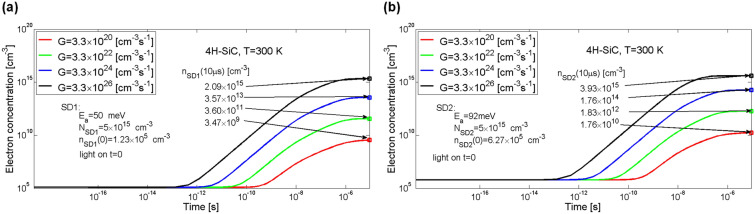
Simulation of temporal changes in electron concentration at trap levels of the shallow donors SD1 (**a**) and SD2 (**b**) in 4H-SiC material. Parameter *G* is the generation rate coefficient.

The following tests are to demonstrate the effect of degree of filling the individual defect levels on changes in concentration of electrons in the conduction band and holes in the valence band. After starting the illumination, an increase in electron concentration at levels SD1 and SD2 can be observed, as shown in Fig. 4a,b, respectively. The increase in the generation rate *G* from 3.3 × 10^20^ to 3.3 × 10^24^ cm^−3^ s^−1^ resulted in a proportional increase in concentration of electrons at levels SD1 and SD2. For *G* = 3.3 × 10^24^ cm^−3^ s^−1^ trap SD1 was filled in 0.6% and trap SD2 in about 3% and for *G* = 3.3 × 10^26^ cm^−3^ s^−1^ the traps were filled in 40% and in more than 77%, respectively. The larger the value of *G* the earlier the increase in electron concentration at the levels SD1 and SD2 can be observed.

**Figure 5:**
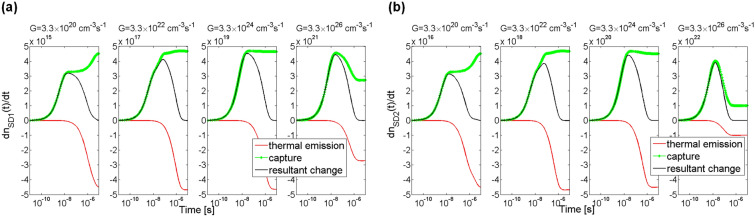
Simulation of rate of change in electron concentration at trap levels SD1 (**a**) and SD2 (**b**) in 4H-SiC material. The green lines show rates of capturing electrons from the conduction band, the red lines show rates of thermal emission of excess electrons from the individual traps to the conduction band, and the black lines are a resultant rate of change in electron concentration as an effect of simultaneous occurrence of the capturing and thermal emission processes. Parameter *G* is the generation rate coefficient.

Additionally, examined was the influence of the generation rate of electron–hole pairs on processes of capturing and thermal emission. The process of capturing electrons from the conduction band by the trap SD1 is shown in Fig. 5a with green lines and the process of thermal emission of electrons from trap SD1 to the conduction band is shown with red lines. The thermal emission process depends on concentration of electrons at trap levels and on the thermal emission rate. As can be seen, the process of capturing electrons from the conduction band starts earlier than the process of thermal emission of electrons from the trap SD1. For each of the assumed generation rates *G*, from the moment of starting the illumination to the time of ca. 10 ns, an increase in capture rates, dependent on concentration of electrons in the conduction band, is visible. Later, the capture rate behaviour is influenced by the generation rate *G*. For two cases of *G* = 3.3 × 10^20^ and *G* = 3.3 × 10^22^ cm^−3^ s^−1^ an increase in capture rate is observed, while for the other two cases of *G* = 3.3 × 10^24^ and *G* = 3.3 × 10^26^ cm^−3^ s^−1^ a decrease is observed.

The processes of thermal emission of electrons from the trap SD1 to the conduction band are visible only after the time of ca. 10 ns. The processes are influenced by electron concentration at the trap level. For each of the assumed generation rates *G* after the time period of a few microseconds the rate of capturing electrons by the trap SD1 is same as the rate of thermal emission of electrons from this trap. Therefore, after this time period, there are no changes in concentration of electrons at trap SD1 and in the conduction band.

Exchange processes of electrons between trap SD2 and the conduction band are very similar to those for trap SD1, which can be seen in Fig. 5b. It’s only worth noting that for the trap SD2 the rates of capture and thermal emission of electrons are one order of magnitude greater than those for trap SD1.

**Figure 6:**
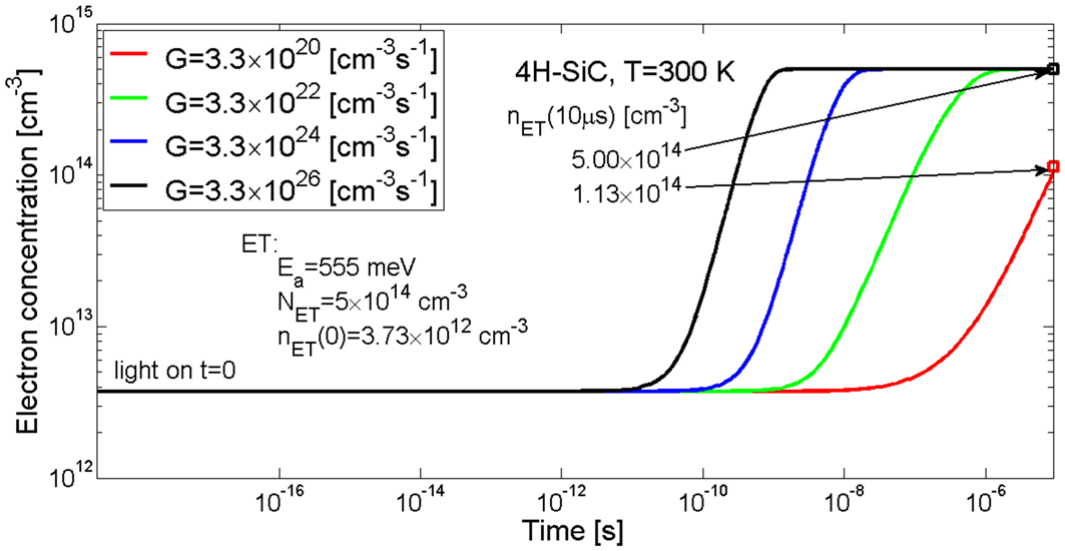
Simulation of the temporal changes in electron concentration at the electron trap level ET in 4H-SiC material. Parameter *G* is the generation rate coefficient.

Simulation results of changes in electron concentration at the trap level ET occurring after starting the illumination is shown in Fig. 6. As can be seen, after the time period of 10 µs electron concentration for *G* = 3.3 × 10^20^ cm^−3^ s^−1^ increased to 1.1 × 10^14^ cm^−3^ and would still be rising. Whereas for the remaining greater values of rate of generation *G* electron concentration also increased, but stabilised at 5 × 10^14^ cm^−3^, which means that the centre is completely filled with the carriers. Greater value of the *G* coefficient caused an earlier increase in the concentration of electrons at the trap ET.

**Figure 7:**
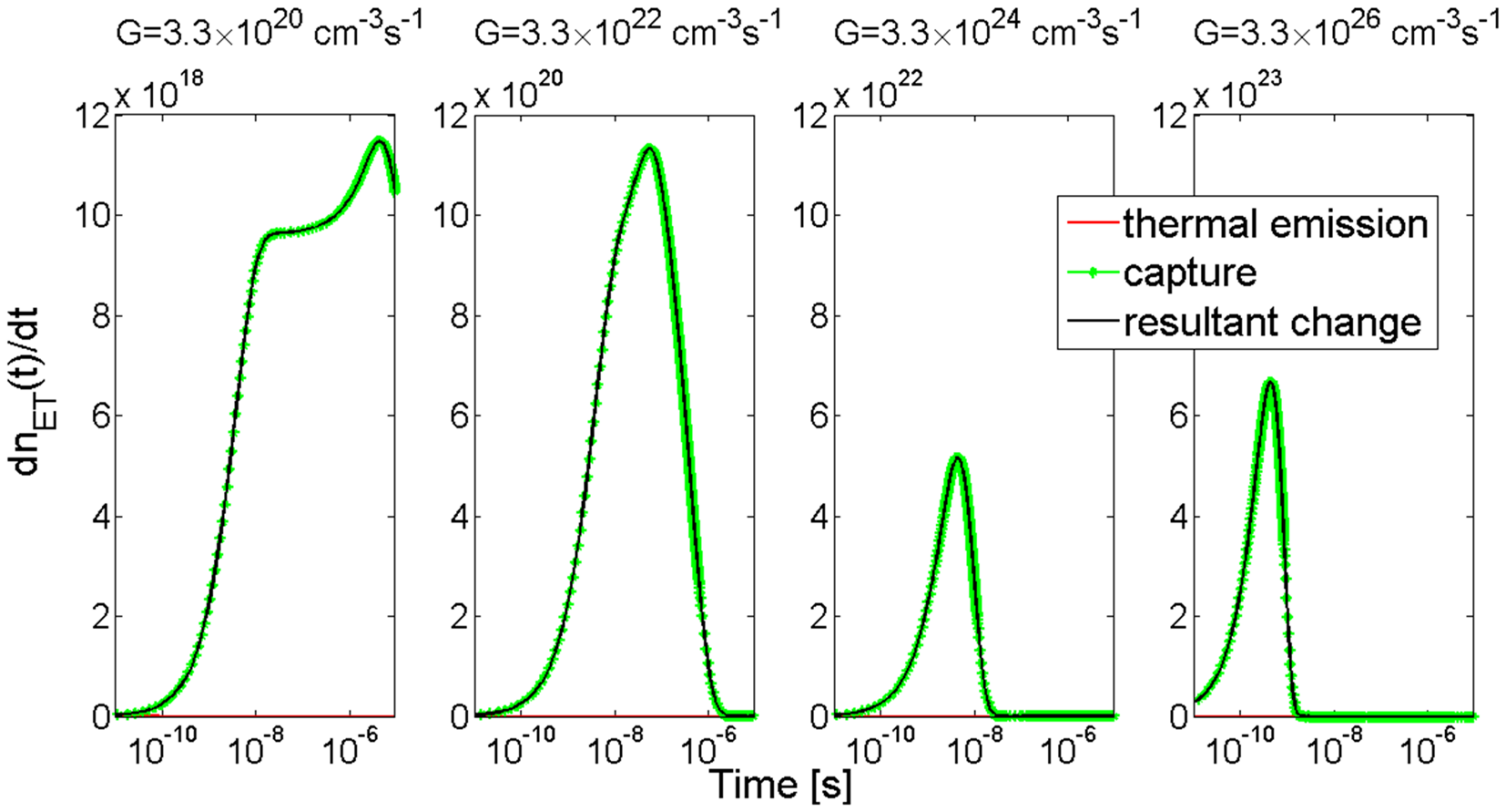
Simulation of rate of change in electron concentration at trap level ET in 4H-SiC material. The green lines show rates of capturing electrons from the conduction band, the red lines show rates of thermal emission of excess electrons from the trap to the conduction band, and the black lines overlaid on the green ones are a resultant rate of change in electron concentration as an effect of simultaneous occurrence of the capturing and thermal emission processes. Parameter *G* is the generation rate coefficient.

In Fig. 7 shown are two processes, i.e. a process of capturing electrons from the conduction band by the trap ET (green lines) and a process of thermal emission of electrons from the trap ET to the conduction band (red lines). As it can be seen, for all the generation rates *G* there are only processes of capturing electrons from the conduction band (no thermal emission observed). The rate of electron capture by the trap first rises and then, after reaching a maximum, falls to zero when the trap level ET is filled up with electrons. However, the zero-rate value is achieved at different times for each of the *G* coefficients. For *G* = 3.3 × 10^22^ cm^−3^ s^−1^ electron capture rate reaches zero after the time of 4 µs, for *G* = 3.3 × 10^24^ cm^−3^ s^−1^ after 40 ns and for *G* = 3.3 × 10^26^ cm^−3^ s^−1^ after 2 ns, whereas for the smallest rate of generation *G* = 3.3 × 10^20^ cm^−3^ s^−1^ the rate of change in electron concentration is small enough that till the time of 10 μs the electron trap was not filled up thus the equilibrium was not settled.

### Impact of the generation rate on the occupation of hole traps in SI 4H-SiC

**Figure 8:**
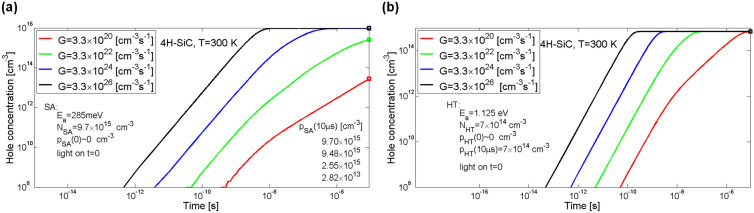
Simulation of the temporal changes in hole concentration at trap levels of the shallow acceptors SA (**a**) and of the deep hole trap HT (**b**) in 4H-SiC material. Parameter *G* is the generation rate coefficient.

Simulation results show that illumination of the 4H-SiC material increases hole concentrations at both trap levels SA and HT as can be seen in Fig. 8. But after the time period of 10 µs the trap level SA was filled with 100% only for the two greatest values of generation rate coefficient *G*, i.e. 3.3 × 10^24^ and 3.3 × 10^26^ cm^−3^ s^−1^. For the other two values of *G*, the trap level SA was not completely filled, and the hole concentration reached a value of 3.91 × 10^13^ cm^−3^ for *G* = 3.3 × 10^20^ cm^−3^ s^−1^ and a value of 3.27 × 10^15^ cm^−3^ for *G* = 3.3 × 10^22^ cm^−3^ s^−1^ (Fig. 8a). In the case of the trap level HT, a complete filling is observed for all values of *G* within a time period of 10 µs (Fig. 8b).

**Figure 9:**
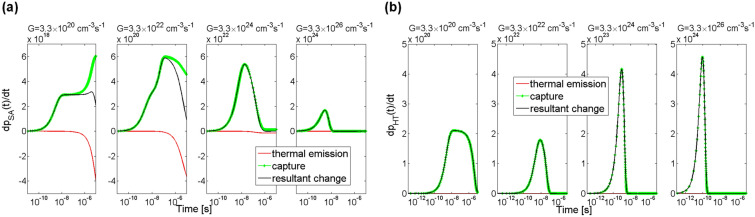
Simulation of the rate of change in hole concentration at trap levels SA (**a**) and HT (**b**) in 4H-SiC material. The green lines show rates of capturing holes from the valence band, the red lines show rates of thermal emission of excess holes from the traps to the valence band, and the black lines overlaid in most cases on the green ones are a resultant rate of change in hole concentration as an effect of simultaneous occurrence of the capturing and thermal emission processes. Parameter *G* is the generation rate coefficient.

In Fig. 9a shown are two processes, i.e. a process of capturing holes from the valence band by the trap SA (green lines) and a process of thermal emission of holes from the trap SA to the valence band (red lines). In this case the thermal emission process depends on concentration of holes at the trap level SA and on the thermal emission rate coefficient for this trap. The processes can be divided into two phases. In the first one, the thermal emission of holes is irrelevant in comparison with the process of capturing holes from the valance band by the trap SA. It reveals as an increase in rate of change in hole concentration at the trap level SA. Only in the second phase, process of thermal emission of holes to the valence band becomes more and more significant. It leads to inhibition of the capture process and consequently to a decrease to zero in the resultant rate of change in hole concentration at the trap level SA, although is not entirely visible in two first cases, i.e. for *G* = 3.3 × 10^20^ and *G* = 3.3 × 10^22^ cm^−3^ s^−1^. Additionally, it can also be seen that the greater the value of the generation rate *G* the earlier a maximum in the resultant rate occur.

Figure 9b presents simulation results of temporal changes in rate of exchanging holes between the valence band and a deep hole trap HT as processes of capture (green lines) and thermal emission (red lines) for various values of the generation rate coefficient *G*. As can be seen, for all the cases there are only processes of capturing holes present. The rate of capturing holes by the trap HT increases and after reaching its maximum, decreases to zero, which means that the trap has been filled up with holes captured from the valence band.

### Impact of the generation rate on the occupation of recombination centre in SI 4H-SiC

**Figure 10:**
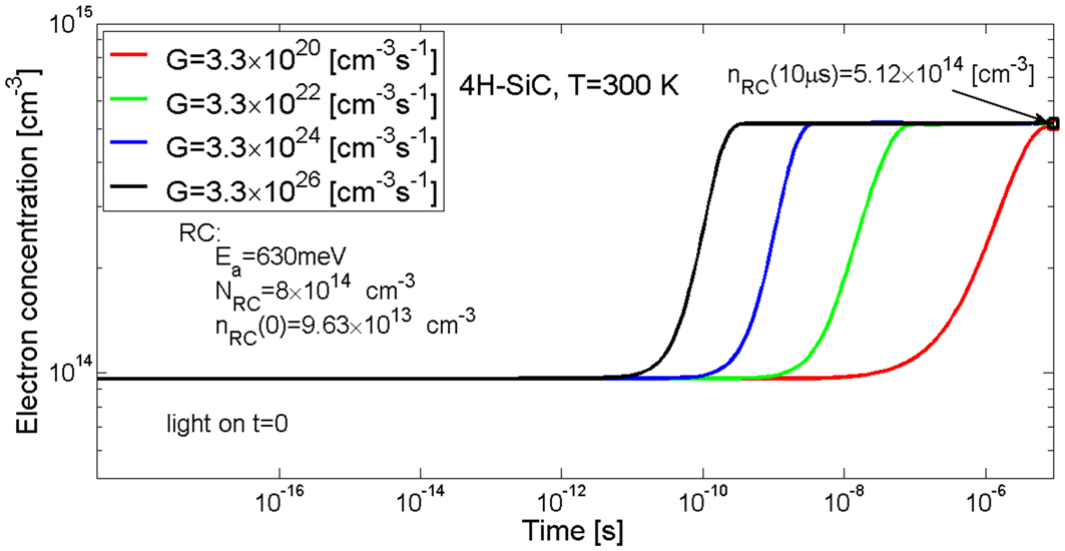
Simulation of the temporal changes in electron concentration at trap level of the recombination centre RC in 4H-SiC material. Parameter *G* is the generation rate coefficient.

Temporal changes in concentration of electrons at the recombination centre level RC as a result of photon illumination is shown in Fig. 10. For each value of the generation rate *G*, within the time period of 10 µs, the electron concentrations set at 4.46 × 10^14^ cm^−3^, what means that the RC level is filled in approx. 55%. Moreover, the greater the value of the generation rate coefficient *G* the earlier the changes in electron concentration begin to occur.

**Figure 11:**
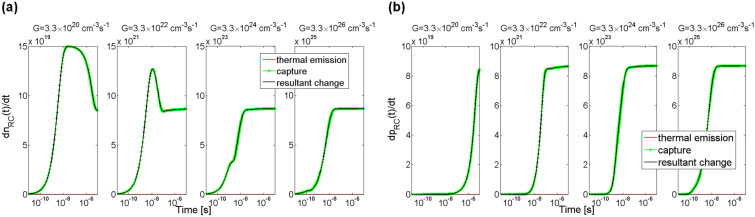
Simulation of the rate of exchange of electrons between the recombination centre and the conduction band (**a**) and of holes between the recombination centre and the valence band (**b**) in 4H-SiC material. The green lines show rates of capturing electrons from the conduction band and holes from the valence band, and the red lines show rates of thermal emission of electrons and holes from the centre. As there is no thermal emission present, a resultant rate of exchange of electrons and holes (black lines) is a consequence of the capturing process itself.

In the case of exchanging electrons between the conduction band and the recombination centre RC (Fig. 11a) only a process of capturing electrons by this centre is visible, the process of thermal emission is not present. It is worth noting that for the two smallest values of the generation rate *G*, i.e. for 3.3 × 10^20^ and for 3.3 × 10^22^ cm^−3^ s^−1^ there are three stages of the rate of change in electron concentration visible. The first one, when the increase in the rate of capturing electrons from the conduction band is observed, begins at the very moment the illumination starts and lasts up to approx. 30 ns for *G* = 3.3 × 10^20^ cm^−3^ s^−1^ and to approx. 15 ns for *G* = 3.3 × 10^22^ cm^−3^ s^−1^. The second stage reveals as a reduction in the rate of capturing electrons by the RC centre which is a result of increase in concentration of electrons at the RC level. In the last stage the rate of electrons capturing is impacted by a concentration of excess electrons in the conduction band.

In the case of exchanging holes between the valence band and the recombination centre RC (Fig. 11b) incident photons activate a process of capturing holes by this centre. The rate of capturing holes is directly proportional to the concentration of holes in the valence band, to the concentration of electrons at the centre’s level, and to the hole capture coefficient associated with the defect centre. In turn, due to a very small value of the thermal emission coefficient (see Table 2), the rate of the thermal emission of holes from the recombination centre is negligible. In the case of the recombination centre RC, the process of capturing holes from the valence band is balanced by the process of capturing electrons from the conduction band.

### Impact of degree of filling of the defect centres on the rate of concentration change of electrons in the conduction band and of holes in the valence band in SI 4H-SiC

It is a summary that illustrate the impact of recombination processes and exchange of charge carriers between the bands and the individual defect centres on the rate of change in concentration of the carriers.

**Figure 12:**
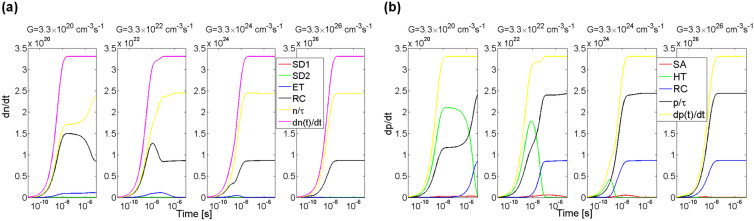
Impact of individual defect centres (SD1, SD2, ET, RC) and the process of recombination of electrons (*n*/τ) on the rate of change in concentration of electrons in the conduction band (**a**) and impact of individual defect centres (SA, HT, RC) and process of recombination of holes (*p*/τ) on the rate of change in concentration of holes in the valence band (**b**) in 4H-SiC material. Shown data includes both the processes of capture and thermal emission of charge carriers. Parameter *G* is the generation rate coefficient. Exchange rates between conduction band and the shallow donor defects SD1 and SD2 is negligible.

In Fig. 12a shown are processes of recombination and exchange of electrons in the conduction band as a rate of change in their concentration in this band. Results of simulations for four values of rate of generation of electron–hole pairs *G* are presented, i.e. for *G* equalled 3.3 × 10^20^, 3.3 × 10^22^, 3.3 × 10^24^ and 3.3 × 10^26^ cm^−3^ s^−1^. As it can be seen, the greatest impact on the rate of change in electron concentration in each of the four cases have the processes of band-to-band recombination, as well as the exchange of electrons with the recombination centre.

Additionally, for all the cases, except for the case of *G* = 3.3 × 10^26^ cm^−3^ s^−1^, a process of capturing electrons from the conduction band by the electron trap ET can be seen. With pink lines denoted was rate of change in electron concentration in the conduction band which is a sum of recombination rate and the rates of exchange of charge carriers between the band and the individual defect centres. It must be noted that the exchange rates with the shallow donor defects SD1 and SD2 is negligible. In the equilibrium state, i.e. after the time period of 10 µs, for each of the four cases, the rate of change in electron concentration in the conduction band is equal to the rate of generation of electron–hole pairs *G*.

In Fig. 12b shown are processes of recombination and exchange of holes in the valence band as a rate of change in their concentration in this band. As usually, results of simulations for four values of rate of generation of electron–hole pairs *G* are presented. Green lines denote a process of exchanging holes between valence band and the deep hole trap defect HT. Red lines denote a process of exchanging holes with a shallow acceptor centre SA. The process of recombination has been shown with black lines and the process of exchanging holes with a recombination centre has been shown with blue lines. Yellow lines denote a sum of the recombination process and the exchange of holes between the valence band and the individual centres. In three of the cases, i.e. for *G* equalled 3.3 × 10^20^, 3.3 × 10^22^ and 3.3 × 10^24^ cm^−3^ s^−1^ the greatest impact on the concentration of holes in the valence band has the process of exchanging holes with the deep hole trap HT. When the rate of capturing holes by the trap HT is decreased, then the processes of hole recombination and exchange of charge carriers with the recombination centre become the most important. Again, in the equilibrium state, for each of the four cases, the rate of change in hole concentration in the valence band is equal to the rate of generation of electron–hole pairs *G*.

## Model verification

Model of the 4H-SiC material presented in the paper has been verified with experimental data. The verification was based on series of measurements of resistivity changes of 4H-SiC sample material under the influence of various optical conditions, i.e. a dark resistivity, and a light resistivity for two slightly different values of photon flux. Changes in resistivity can be observed as changes in photoconductivity determined from measurements of photocurrents induced by a flux of incident light of energy greater than the material's band gap. The measurements has been conducted in Lukasiewicz Research Center—Institute of Electronic Materials Technology by Prof. Kaminski's research team. The 4H-SiC material was a wafer with dimensions of 4 × 9 × 0.38 mm on top of which made were two ohmic contacts with dimensions of 2.5 × 2.5 mm separated by a 0.7-mm gap.

**Figure 13:**
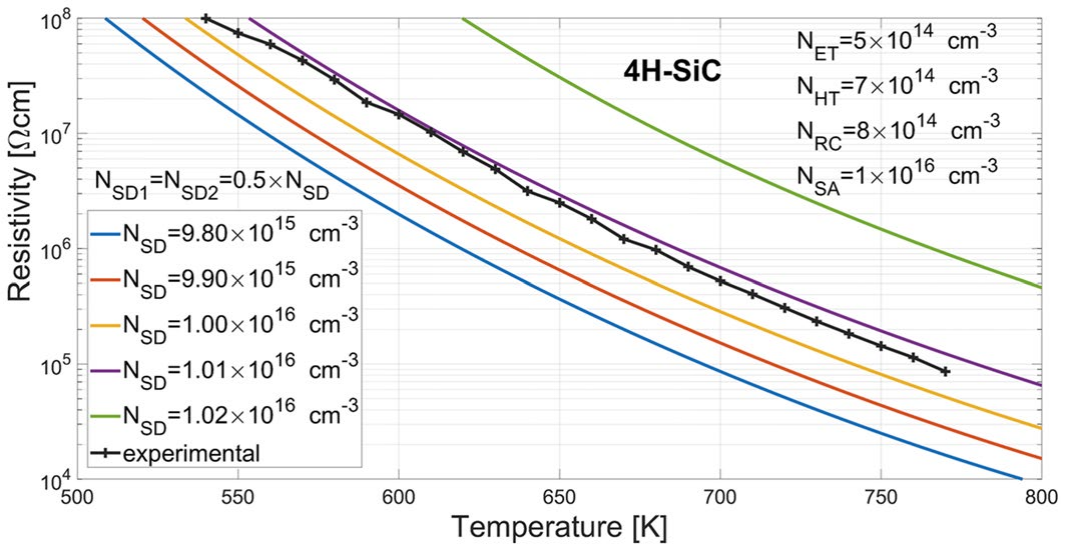
Comparison results of measurements and simulations of dark resistivity in a range of temperature from 540 to 770 K at 10 K steps.

The dark resistivity has been both experimentally determined and simulated in a temperature range of 540–770 K at 10 K steps and the results have been compared as shown in Fig. 13. The experimental determination was based on dark current amplified and measured with Keithley 428 picoammeter. The current was provided by a source voltage of 30 V applied to the ohmic contacts of the sample material put successively in mentioned temperatures. The resistivity was then obtained by using the fundamental formula ρ = *R*·*S*/*L*, where *R* is a ratio of the source voltage and the measured dark current, *L* is a distance between the ohmic contacts, and *S* is a cross-sectional area specified as a product of ohmic contact width and wafer thickness.

The simulations have been conducted for the assumed defect parameters of the 4H-SiC model (*N*_*ET*_ = 5.00 × 10^14^ cm^−3^, *N*_*HT*_ = 7.00 × 10^14^ cm^−3^, *N*_*RC*_ = 8.00 × 10^14^ cm^−3^, *N*_*SA*_ = 1.00 × 10^16^ cm^−3^, *N*_*SD*_ = 1.00 × 10^16^ cm^−3^, wherein *N*_*SD*1_ = *N*_*SD*2_ = 0.5 × *N*_*SD*_), as well as for four additional slightly different values of shallow donor concentrations *N*_*SD*_ = (0.98 × 10^16^, 0.99 × 10^16^, 1.01 × 10^16^, 1.02 × 10^16^) [cm^−3^]. As it can be seen, the simulated dark resistivity matches the experimental one in a wide temperature range. Although the simulated resistivity for *N*_*SD*_ = 1.01 × 10^16^ is better fitted to the experimental data than the one for the assumed value of *N*_*SD*_ = 1.00 × 10^16^, our model of the 4H-SiC material can still be considered well fitted to the physical semiconductor material as the measurement errors of defect concentrations themselves are well above the difference between 1.01 × 10^16^ and 1.00 × 10^16^.

**Figure 14:**
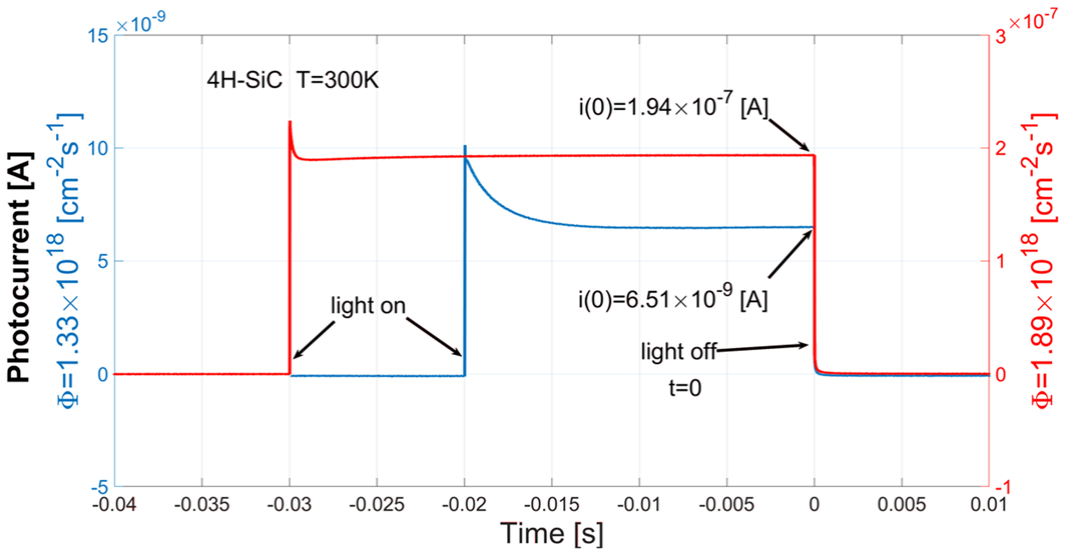
Photocurrent pulse waveforms induced by an incident light with a wavelength of 375 nm for two different values of photon flux: Φ = 1.33 × 10^18^ cm^−2^ s^−1^ (blue), and Φ = 1.89 × 10^18^ cm^−2^ s^−1^ (red).

The experimental light resistivity was determined from measurements of photocurrent pulses induced with a UV semiconductor laser light with a wavelength of 375 nm (3.31 eV). The pulses' duration was 20 or 30 ms, and the repetition period was 500 ms. The photocurrent transients, provided by a source voltage of 30 V applied to the ohmic contacts of the sample material put at a temperature of 300 K, again, were amplified and measured with Keithley 428 picoammeter. The measurements were digitized with a 12-bit amplitude resolution and a 10-µs time resolution, and then 250 digital pulse waveform data were averaged to improve the signal to noise ratio. For the photon flux Φ = 1.33 × 10^18^ cm^−2^ s^−1^ the steady-state photocurrent value was equal to 6.51 × 10^–9^ A, and for the photon flux Φ = 1.89 × 10^18^ cm^−2^ s^−1^ the photocurrent value was equal to 1.94 × 10^–7^ A (Fig. 14). The light resistivity has been obtained based on the measured steady-state photocurrent, and, again, source voltage of 30 V, and geometrical dimensions of the sample material.

In order to determine the simulated light resistivity, transients of charge carrier concentration in conduction and valence bands for both the photon flux values, i.e. Φ = 1.33 × 10^18^ cm^−2^ s^−1^ and Φ = 1.89 × 10^18^ cm^−2^ s^−1^, were computed and the transients' steady-state values were determined. Finally, the simulated resistivities were obtained as the reciprocal of conductivity described by (5).

Comparison of the experimental and simulated light resistivities determined for both photon flux values are shown in Table 3. As the results show, the experimental and simulated conductivities are of the same orders of magnitude which proves that our model of 4H-SiC material corresponds to the actual physical 4H-SiC sample.

**Table 3:** Resistivity measurements and simulations of illuminated 4H-SiC material for two photon flux values.

Photon flux [cm^−2^ s^−1^]	Resistivity [Ω cm]	
	Experimental	Simulated
1.33 × 10^18^	6.42 × 10^5^	1.16 × 10^5^
1.89 × 10^18^	1.84 × 10^4^	4.63 × 10^4^

## Conclusions

The paper presents effect of rate of generation of electron–hole pairs on changes in conductivity of semi-insulating 4H-SiC semiconductor material. The study was conducted for a model assuming two shallow donors, hole and electron traps, an acceptor and a recombination centre. Calculations were performed with the help of a simulator developed for the aim of the study. Its task was to solve a system of differential equations describing change in concentration of charge carriers in the valence and conduction bands, and those captured by the modelled defect centres. The equations considered transitions of charge carriers between the bands and the defect levels, as well as band-to-band recombination processes and a recombination via the recombination centre.

Results of the simulations show that changes in conductivity of the SI 4H-SiC obviously are dependent on electron–hole pairs generation rate. The greater the generation rate the earlier the conductivity starts to change (rise) and the less time it requires to achieve a certain level of conductivity, which can be interpreted as the shorter turn-on time of a hypothetical photoconductive switch. Conductivity reaches its maximum and stops changing after the period of approx. 10^–8^ s, irrespective of the assumed generation rates. The time frame corresponds to the lifetime of charge carriers assumed for the simulation. Since the concentration of electrons and holes for the same excitation is identical, electron mobility has a decisive impact on changes in the conductivity.

The rate of increase in the concentration of excess charge carriers depends on both the lifetime of the carriers and the generation rate of electron–hole pairs. The latter also has an influence on the rate of capture of excess charge carriers by electron and hole traps as well as on a degree of filling the traps with charge carriers at the equilibrium state. The greatest impact on changes in electron concentration in conduction band have two types of recombination processes, band-to-band recombination and recombination via recombination centre RC. In the case of holes, their concentration in valence band depends mainly on the recombination processes, but for the generation rates *G* = 3.3 × 10^20^ and *G* = 3.3 × 10^22^ cm^−3^ s^−1^, processes of capture by the hole traps HT can also be seen.

Presentation of the changes in concentration of charge carriers at a logarithmic timescale, allows observing in detail the processes of capturing and emission of charge carriers from the assumed energy levels occurring immediately after photon illumination was applied. Analysis of rapid changes in conductance of the 4H-SiC semiconductor can be particularly useful in assessing switching frequency of photoconductive semiconductor switches and thus aiding their design process.

## Data Availability

The datasets generated and analysed during the current study are available from the first author on reasonable request.
